# Methods and metrics challenges of delivery-system research

**DOI:** 10.1186/1748-5908-7-15

**Published:** 2012-03-12

**Authors:** Jeffrey A Alexander, Larry R Hearld

**Affiliations:** 1 School of Public Health, Department of Health Management and Policy, The University of Michigan, Ann Arbor, MI, USA; 2 School of Health Professions, Department of Health Services Administration, University of Alabama at Birmingham, Birmingham, AL, USA

## Abstract

**Background:**

Many delivery-system interventions are fundamentally about change in social systems (both planned and unplanned). This systems perspective raises a number of methodological challenges for studying the effects of delivery-system change--particularly for answering questions related to whether the change will work under different conditions and how the change is integrated (or not) into the operating context of the delivery system.

**Methods:**

The purpose of this paper is to describe the methodological and measurement challenges posed by five key issues in delivery-system research: (1) modeling intervention context; (2) measuring readiness for change; (3) assessing intervention fidelity and sustainability; (4) assessing complex, multicomponent interventions; and (5) incorporating time in delivery-system models to discuss recommendations for addressing these issues. For each issue, we provide recommendations for how research may be designed and implemented to overcome these challenges.

**Results and conclusions:**

We suggest that a more refined understanding of the mechanisms underlying delivery-system interventions (treatment theory) and the ways in which outcomes for different classes of individuals change over time are fundamental starting points for capturing the heterogeneity in samples of individuals exposed to delivery-system interventions. To support the research recommendations outlined in this paper and to advance understanding of the "why" and "how" questions of delivery-system change and their effects, funding agencies should consider supporting studies with larger organizational sample sizes; longer duration; and nontraditional, mixed-methods designs.

A version of this paper was prepared under contract with the Agency for Healthcare Research and Quality (AHRQ), US Department of Health and Human Services for presentation and discussion at a meeting on "The Challenge and Promise of Delivery System Research," held in Sterling, VA, on February 16-17, 2011. The opinions in the paper are those of the author and do not represent the views or recommendations of AHRQ or the US Department of Health and Human Services.^1^

## Background

It is increasingly evident that patient outcomes are not solely a function of efficacious clinical interventions and practices. In its 2009 Report to the President and the Congress, the Federal Coordinating Council for Comparative Effectiveness Research (FCC) noted that research to date "has been disproportionately focused on pharmacologic treatments rather than the full spectrum of intervention types," and that there is a need for rigorous demonstrations and evaluations that will show which delivery-system designs and improvement approaches are most effective, under what circumstances, and for whom--and what it would take to replicate or scale up such approaches.

Delivery-system research may be viewed as the systematic study of healthcare organizations, including interchanges with their external environments (e.g., markets, regulators, competitors) and interactions among internal components (e.g., employees, technology, work processes, culture), that affect how care is organized and provided [1]. The goal of delivery-system research is to use research evidence to systematically identify which system processes, structures, or strategies are most effective for improving outcomes for patients and to use such evidence as the basis for implementing interventions and formulating policy to shift care to these value-maximizing options across the healthcare system. However, unlike pharmacologic interventions, which can be controlled in experimental designs, evaluating the effectiveness of different approaches to delivering care poses challenges to many of the traditional tenants of designing and conducting research.

This claim stems from the observation that many delivery-system interventions and innovations are fundamentally about change in social systems (both planned and unplanned). These changes occur within a broader social context and involve interactions and relationships among actors, stakeholders, market conditions, historical and cultural milieus, etc. The fidelity with which a given intervention is implemented and practiced by a particular provider or group of providers follows therefore from interrelationships among a range of internal and external factors that constitute the social system surrounding the intervention. This systems approach is cited as pivotal to understanding (and solving) a number of quality and safety problems identified in publications such as *To Err is Human *and *Crossing the Quality Chasm *[2,3]. These publications encourage health-services researchers to consider greater application of systems-focused theory to questions of how organizational factors shape quality and other patient-related outcomes.

As noted earlier, a systems perspective raises a number of methodological challenges for those interested in studying the effects of delivery-system interventions, particularly in employing methods dictated by traditional experimental and quasi-experimental designs. Indeed, others have noted that "experimentalists have pursued too single-mindedly the question of whether a [social] program works, at the expense of knowing why it works" [4]. Similarly, Berwick states that "... although the [traditional experimental] model seeks generalizable knowledge, in that pursuit it relies on--it depends on--removing most of the local details about 'how' something works and about the 'what' of contexts" [5].

In this paper, we discuss the research challenges posed by five key methods and metrics issues in delivery-system research: (1) modeling intervention context; (2) measuring readiness for change; (3) assessing intervention fidelity and sustainability; (4) assessing complex, multicomponent interventions; and (5) incorporating time in delivery-system models. We focus on these particular issues because, from a systems perspective, they are related to a core set of interdependent components that contribute to or compromise the effectiveness of healthcare interventions [6]. According to this perspective, systems behave according to a number of key properties:

1. Each component can affect the behavior or properties of the whole system.

2. Each component is necessary but is not sufficient to achieve the objectives or functions of the system.

3. Behavior and properties of one component depend on the behavior of other parts of the system.

**Figure 1:**
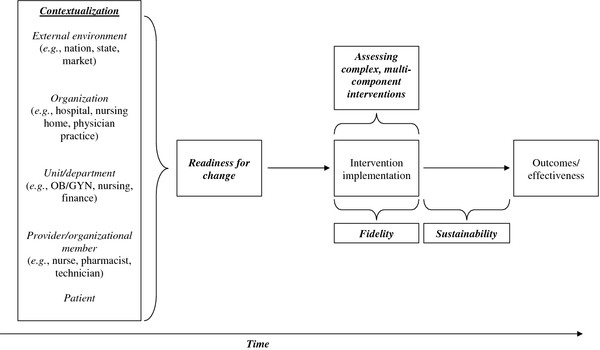
**Method and metric challenges associated with studying delivery-system change^a^**.

Healthcare organizations and the implementation of delivery-system interventions within them exhibit these properties [2,3]. Figure 1 provides an overview of the methodological and metric issues associated with studying delivery-system interventions implemented in complex social systems.

In subsequent sections, for each of the five issues noted above, we first provide background about the issue, including the methodological challenges posed by each issue, followed by specific strategies for how investigators can address these challenges in their own research (Table 1). Although some of these specific issues have been noted by others [7-10], our discussion is intentionally broader to illustrate the range of the methods and metrics challenges faced by delivery-system researchers and the relationships between them. We conclude with comments on the interconnections between these issues and more general recommendations of what will be required collectively to make the next generation of delivery-system research successful.

**Table 1:** Challenges and recommendations for delivery-system research

Issue	Challenge	Recommendation	Examples
Modeling intervention context	• Delivery-system intervention may be mediated by a range of contextual features (e.g., human, sociocultural, and organizational factors) that may accentuate or attenuate its effect on patient care outcomes	• Contextualization through detailed description and informed reflection on the role that context plays in influencing the meaning, variation, and relationship among variables under study • Use multilevel modeling to assess the influence of context on processes at lower levels (e.g., state, organization, team)	Brooks et al. [11] conducted a comparative study using qualitative and case study data-collection methods, including semistructured interviews with key stakeholders and follow-up telephone interviews over a one-year period, to identify contextual influences inhibiting or promoting the acceptance and integration of innovations in mental health services in both National Health Service (NHS) and community settings.

Readiness for change	• Not all organizations or providers willing or able to undertake change Confounding capacity for change and readiness for change	• Systematically assess readiness for change prior to evaluation of delivery-system change Develop separate measures and assessment of readiness and capacity for change	Based on survey data from 249 drug treatment units, Fuller et al. [12] assessed four aspects of readiness for change (motivation for change, institutional resources, staff attributes that influence organizational change, and organizational climate) and found that units with higher levels of readiness for change were associated with workers who had more positive opinions about the use of evidence-based treatment.

Assessing intervention fidelity and sustainability	• Dynamic social context increases the risk of delivery-system intervention deviating from its intended form • Cross-sectional/short study durations make determination of long-term effects/changes difficult to assess • Changes may experience entropy and/or revert back to established routines and practices	• Implementation monitoring to assess the degree to which new structures and practices have been deployed • Focus on group, organizational, or external factors rather than more common individual attitudes • Design and measure multiple factors that may influence intervention implementation (e.g., resources, prior experience with similar changes) • Longitudinally assess key program elements	Orwin [13] used quarterly reporting forms, site visits, and bimonthly telephone calls to construct implementation histories of a substance abuse program. These implementation histories were then compared to the logic model of the original, proposed program to assess whether interventions were implemented as planned. The study also used surveys of program participants to construct a fidelity measure that assessed the degree to which the services that were intended to be delivered to all or most participants were in fact delivered.

Assessing complex, multicomponent interventions	• Difficult to parse out the effects of individual intervention components and determine whether some components are more important than others • Measures of intervention effects assumed to be linear and additive	• Complement traditional quantitative methods with qualitative methods (i.e., multimethod designs) to assess dynamic, multifaceted aspects of complex delivery-system interventions	English et al. [14] used a multimethod design (interviews, group discussions, field notes, detailed longitudinal quantitative data) to examine reasons why an intervention intended to improve essential pediatric hospital services in Kenya did (or did not) produce its desired effects.
Incorporating time as an analytic variable in delivery-system research	• Short evaluation periods make determination of long-term effects/changes difficult to assess • Patients/organizations may experience different rates and directions of change over time Effects of intervention may differ over time	• Identify and longitudinally monitor key elements of intervention • Incorporate temporal aspects of the intervention-patient outcome(s) relationship into conceptual and empirical models • Identify temporal patterns in the data • Directly assess time by including time-varying predictors • Examine interactions among interventions variables and patient growth trajectories	Brekke et al. [15] used linear growth models to assess whether prospective client outcomes over a 36-month period varied across (i.e., had different trajectories) three types of community-based, psychosocial rehabilitation programs for individuals with chronic mental illness.

## Discussion

### Modeling intervention context

#### Background and challenges

The impact of delivery-system interventions on patient outcomes is potentially mediated by a range of human, sociocultural, and organizational factors collectively referred to as context, or more formally defined as the situational opportunities and constraints that affect the occurrence and meaning of organizational activities [16,17]. Organizations in which delivery-system changes occur, for example, may exhibit considerable variability in terms of structure, mission, resource availability, and staff support over a given time period that may facilitate or impede the effective use of an intervention. Minimally, variation in facilitators and barriers necessitates examination of initial variability across organizations to understand the course of development and response to an intervention. However, unless context is formally incorporated in models of delivery-system change, this baseline assessment of contextual variability may not be sufficient to address threats to internal and external validity that may result from interactions between the delivery-system intervention and contextual factors in settings to which one might wish to generalize. For example, patient-centered medical home (PCMH) initiatives typically involve substantial changes in clinical and administrative practices that require significant resource investments [18]. Yet, physician practices exhibit considerable variability in their capacity to commit and sustain the level of resources needed to make these changes. Failure to account for these differences in the study design, especially in the case of null findings, makes it difficult to determine whether nonsignificant effects of PCMH on patient outcomes are due to an ineffective intervention or insufficient capacity to implement the PCMH in the practice setting.

Because context shapes implementation opportunities and constraints, it can increase or decrease variation in outcomes or interventions of interest [16]. This impact of context is important because it can result in interventions displaying different strengths, causal directions, and base rates depending on the ecological conditions under which the processes or programs are observed [17]. For instance, some research suggests that physician practices with more access to resources may experience higher base rates of PCMH implementation because they are able to dedicate more time and effort to prepare the practice for change [18]. In contrast, physician practices with limited access to resources (e.g., small physician practices) may experience lower base rates of change without the resources needed to lay the groundwork for implementation. To date, most treatments of context in delivery-system research have been limited to cursory descriptions of a particular context, stating as a study limitation that context may affect generalizability. The challenge for researchers is to conduct more systematic analyses of organizational context. Here, *systematic *implies that the context of the study is theorized as a conceptual construct, operationalized as a variable in the study, and that variance associated with the context is directly incorporated in the analysis [19].

Additionally, given the nested dependencies of factors that make up context, it typically consists of multiple levels. In this ecological view, *levels *are the various contextual layers, such as the national and state health policy environment, organization, healthcare provider team, family, and individual patient characteristics, that may directly or indirectly influence a range of health outcomes. Each successive contextual level may influence and be influenced by adjacent or nonadjacent levels. Actors may interact with one another *within *and *between *each contextual level. Because of the complexity of the potential effects of factors within each contextual level on behavior, analysis of their influences is challenging.

### Recommendations: Contextualizing delivery-system research

Contextualization is the process whereby knowledge of the settings to be studied is brought to bear in conceptualization, research design, and implementation decisions [19]. Whereas direct measurement and analysis of individual or bundled contextual effects are probably the most intuitive to most delivery-system researchers, other approaches may also contribute to understanding the role of context. Indeed, in some cases qualitative methods such as participant observation and archival document analysis may provide insights into context that simply are not possible or practically feasible via direct measurement methods such as surveys. Likewise, key informant interviews can provide detailed description and informed reflection on the role that context plays in influencing the meaning, variation, and relationship among variables under study. Such approaches may be appropriate when contextual variation is limited by the sample and/or when key contextual factors (e.g., history) cannot be quantified.

Comparative analyses of delivery-system interventions across different contexts can also be a powerful method of assessing contextual effects, even with relatively few comparative observations [20]. For example, the phased approach to health-reform implementation in the United States and the progressive release of details about different components of this legislation (e.g., accountable care organizations [ACOs]) potentially affect implementation decisions by provider organizations, yet present a number of challenges to quantitatively measuring how contextual factors affect the decision-making process and care processes that stem from such decisions. In the case of ACOs, earl- adopting provider organizations looking to gain competitive advantage are likely to be making decisions with limited information about the rules and regulations, which may result in more variation among these organizations with respect to ACO structure, strategy, and governance. In contrast, late-adopting provider organizations are likely to have more complete information, potentially reducing variation among these organizations with respect to these same features. Detailed description of the context of these decision-making processes (e.g., timing of decision, information availability) and a comparison of how these different contexts influenced decisions about ACOs is likely to be important for understanding differences in implementation across organizations and time and how these differences affect patient outcomes. Regardless of the approach, and given the unlimited boundaries of context, it is imperative that delivery-system research employ careful conceptualization of context as it applies to the problem under investigation.

Assuming that context can be directly measured and quantified, an important strategy for analyzing contextual effects is multilevel modeling. Multilevel statistical methods allow analysts to quantify the relative contributions of multiple contextual levels. For example, hierarchical linear models (HLM) offer a powerful approach to conduct longitudinal analyses across three or more levels [21]. These models are also commonly known by other names, such as mixed-effects regression models and multilevel models [22,23]. These approaches are closely related and share several key features. Most importantly, each recognizes the nested or clustered data structures underlying longitudinal and multilevel assessments and introduces adjustments to control for these dependencies. In addition, these approaches permit the inclusion of time-varying as well as time-invariant (i.e., nonchanging, or fixed) covariates. They also recognize the importance of modeling correlated errors when dealing with clustered observations.

In general, these analytic approaches offer the following advantages in delivery-system research: statistically efficient estimates of regression coefficients; use of clustering information to provide correct standard errors, confidence intervals, and significance tests; allowance for uneven assessments and different program tenures (for longitudinal studies); and measurement at multiple levels of a system, which enables examination of whether differences in average outcomes between organizations are explained by contextual factors such as organizational practices/structures or other characteristics of individual patients or providers. Another advantage, one enabled by explicitly considering multiple levels of context, is the examination of cross-level interactions. Cross-level interactions help determine whether contextual effects at lower levels of analysis (e.g., provider team) are consistent in direction and magnitude across higher contextual levels (e.g., unit, organization). This type of analysis is important for identifying contextual factors that may attenuate or strengthen the effects of an intervention in certain circumstances and account for different effects in different settings.

### Readiness for change

#### Background and challenges

Another important consideration for delivery-system research is evaluating readiness for change, or "the extent to which organizational members are psychologically and behaviorally prepared to implement organizational change" [24]. Change in a social context is problematic at best and subject to outright resistance at worst. Put simply, not all organizations or groups are equally good candidates for delivery-system change. For example, a recent study found substantial variation in physician practices' "adaptive reserve," or their ability to cope with changes being introduced by the implementation of a PCMH demonstration program [18,25]. These variations, in turn, had significant implications for the PCMH development trajectory experienced by each practice.

Readiness for change is thus a critical precursor to the successful implementation of complex changes in healthcare settings. While there is considerable conceptual ambiguity about the precise meaning of readiness for change, organizational readiness may be viewed as a supra-individual state of affairs in an organization that reflects organizational members' shared commitment to a specific, designed change and their perceived efficacy to implement the change [24]. Yet, despite the enthusiasm to rapidly pilot and implement delivery-system change among policy makers and national healthcare thought leaders, there has been relatively little attention paid to whether delivery organizations are prepared to take on such transformational change. Indeed, without an understanding and measurement of an organization's, team's, or system's readiness for change and knowledge of successful strategies to increase readiness, change implementation is likely to be hit or miss at best.

A frequent problem in delivery-system research is equating readiness for change with an organization's capacity to undertake and support change. The two are often confounded, but doing so may lead to serious problems of inference. The former is a collective psychological state referring to the cognitive precursors to acceptance or resistance to change, while the latter is usually expressed in structural terms and includes factors such as a delivery system's financial, material, human, and informational resources necessary to support the introduction, routinization, and sustainability of a new practice. The two concepts are related insofar as capacity may shape collective perceptions of efficacy about change, and both need to be considered in delivery-system research.

### Recommendations: Measuring readiness for change

Readiness for change can be measured on two separate, but potentially related, dimensions: motivation and capability [24]. Motivation is the willingness and commitment of organizational members collectively to implement designed organizational change. Capability is organizational members' perceived ability to act on change, or the degree to which organizational members feel that they can be effective in implementing designed change. Readiness for change is highest not only when organizational members want to implement an organizational change but also when they feel confident that they can do so practically. Therefore, as a precursor to any evaluation of delivery-system change, systematic assessment of readiness for change via survey or qualitative assessment may be useful. In such assessments, it is important that investigators go beyond resource issues such as funding, information technology, or technical support (capacity) and focus on the collective psychology of members of the delivery system who will be affected by or asked to implement the change.

Capacity for change is even less well developed, both as a construct and as a measure. Whereas readiness implies a substantive and temporal focus on members of a system prior to introduction of a delivery-system change, capacity implies not only a focus on the implementation phase but ongoing support for the new practice. Investigators should exercise caution about conflating readiness and capacity, conceptually and analytically.

### Assessing intervention fidelity and sustainability

#### Background and challenges

Intervention fidelity refers to whether an intervention was delivered as intended and according to the treatment theory and goals that underlie the intervention [26]. Unlike drug trials or tests of new clinical procedures, the risk of a delivery-system intervention deviating from its intended form is high, given the potentially dynamic social context in which such interventions are introduced and operate. Because many experimental and quasi-experimental approaches treat delivery-system interventions as indivisible, holistic phenomena, program-effectiveness studies often produce conflicting or null findings. In response to criticism levied against so-called "black box" program evaluation studies, evaluators are now more closely attending to the measurement of intervention fidelity and sustainability. The measurement of intervention fidelity provides a means for determining whether key program components were implemented as specified by the program logic model/theory [26,27]. Implementation monitoring allows the evaluator to uncover service delivery breakdowns and unwanted side effects during the early stages of an intervention or entropy of critical program elements over longer periods. Both modes of analysis recognize the potential for real-time process corrections or adjustments [28]. This stands in marked contrast to more traditional approaches to delivery-system research in at least two respects. First, traditional experimental or quasi-experimental approaches assume the intervention operates in a steady-state mode. This allows the researcher to assume that everything is held constant except for his/her ability to control the presence or absence of the intervention--a key requirement for assessing internal validity. Assuming that the intervention or the conditions under which it operates are dynamic and subject to change violates this assumption. Second, the use of research data to inform changes in the intervention itself runs counter to traditional notions of detached objectivity and separation of the investigator from the object of investigation.

Sustainability refers to whether intervention components are active long enough to produce the desired effect on individual patients [29]. Perhaps the most serious and common methodological problem in delivery-system research is short study duration. Implementation of most interventions is measured shortly after the introduction of the intervention or change (typically less than a year, sometimes less than a month), thus making it difficult to tell if the system changes are sustainable over a protracted period. Many changes have short half-lives, and organizations often revert to established routines or practices once the stimulus for a new intervention or change has been removed. Given the tendency for system changes to experience both entropy and reversion to previous states, this is not a trivial matter. Short-term studies or studies that measure implementation at one point in time simply do not provide all the requisite information needed to ascertain whether the changes are going to have long-term effects in organizational settings.

### Recommendations: Measuring and evaluating fidelity and sustainability

Given the systemic nature of delivery-system change and the complexity of implementing such programs, it is critical to assess the degree to which new structures and practices have been deployed in their intended form in order to evaluate their relationship to quality of care and other outcomes. Though implementation research has made significant strides, there remain substantial gaps in the methods used to identify the factors that facilitate or impede efforts to implement change in healthcare organizations. Close monitoring of the implementation process should be incorporated into research designs, especially when program or treatment fidelity data are used to identify the conditions under which treatment outcomes obtain, such as those occurring at the individual patient level. This recommendation stems from recognition that the fidelity with which a given intervention is implemented by a particular provider follows from interrelationships among a range of internal and external factors that constitute the social system that surrounds the intervention. Treatment providers can be expected to systematically increase or decrease adherence to protocol on the basis of a variety of factors, including initial and ongoing training, perceived efficacy, and organizational capacity/support.

Three related recommendations are noted. First, approaches to measuring implementation should focus on group, organizational, or external factors--in addition to the more commonly studied individual attributes and attitudes. This requirement includes adequately accounting for organizational context as a key variable in implementation efforts rather than simply as descriptions of study settings. Second, research design and measurement approaches to implementation need to go beyond assessing the elements of the intervention/program itself and attempt to account for multiple integrative factors that may influence implementation of delivery-system change. These factors might include, for example, resources, leadership, and prior experience with similar changes. Finally, evaluating sustainability of a delivery-system change will require assessment of intervention fidelity over time. Specifically, it cannot be assumed that measuring implementation of a delivery-system change at a single point in time indicates that it will remain stable and/or that reversals will not subsequently occur. Longitudinal assessments of the key program elements are essential to explaining the extent to which a particular delivery-system change has been incorporated into the standard operating practice of a system and, therefore, its potential for ongoing impact on patient outcomes. Assessments of treatment fidelity over time also allow for the possibility of organizational learning, a process by which organizations draw on their experience with the intervention to make adaptive modifications that improve the fit of the intervention to the local context in which it operates.

### Assessing complex, multicomponent interventions

#### Background and challenges

Many delivery-system studies incorporate either multiple interventions or interventions with multiple components. One interpretation is that multiple intervention studies are a reflection of the systemic properties of delivery systems and the emphasis on multifaceted strategies to improve patient-related outcomes. Such studies, however, present important challenges for researchers, insofar as study designs must be able to assess not only how the combined effects of multiple interventions affect outcomes but also assess how, and the extent to which, individual components of the intervention contribute to these collective efforts. However, from a practical standpoint, parsing out the individual effects of multifaceted strategies is often not possible and perhaps even antithetical to the notion of a systems-based approach to quality improvement. That is, highly interdependent components of a complex system may not lend themselves to empirical separation because complex interactions between different components of such systems may produce results that are lost when the cumulative effect is disaggregated into its component parts.

A second, related issue is that most measures of delivery-system interventions are assumed to be linear and additive. The more components of the system in place, the better or more effective the system is assumed to be. However, the underlying dynamics of most intervention measures are difficult to capture simply by checking the presence or absence of specific structural or functional attributes. These may be necessary, but insufficient, to assess whether or not organizations or practitioners are engaged in the intervention or change.

### Recommendations: Using mixed-method research

Despite advances in quantitative methods such as hierarchical linear modeling, there are important questions and concepts in delivery-system research that are not well suited to quantification and which call for a combination of qualitative and quantitative data analyses. For example, among hospital CEOs, organizational processes--such as effective communications, strong leadership, and trust building--appear to play a prominent role in improving quality and other patient outcomes [30]. However, many studies of delivery systems tend to emphasize structural properties (such as size, system affiliation, and ownership), rather than management and team processes, and existing databases usually lack measures or indicators of these complex processes. To the extent that nonlinear and interactive processes and system dynamics are, in fact, important drivers of patient outcomes, delivery-system researchers may be well served by complementing the traditional focus on structural correlates of outcomes with intensive, qualitative research on management processes conducted in smaller samples of organizations.

Despite their intuitive appeal and potential utility in delivery-system research, mixed-methods designs are often misunderstood and difficult to implement in a manner that creates synergistic benefits from the use of different forms of data collection and analysis. Researchers who choose to conduct mixed-methods explanatory studies must consider issues such as the priority or weight given to the quantitative and qualitative data collection and analysis in the study, the sequence of the data collection and analysis, and the stages in the research process at which the quantitative and qualitative phases are connected and the results are integrated [31-33].

If the goal of a mixed-methods approach is to enhance explanation, research designs can take several forms, including but not restricted to (1) connecting quantitative and qualitative phases of the study through selecting participants for the second data-gathering phase based on the findings from the first phase, (2) developing qualitative data-collection protocols grounded in the results of the statistical tests (or vice versa), or (3) integrating quantitative and qualitative results for purposes of interpreting study results and drawing implications for policy or practice. An integrative strategy for combining quantitative and qualitative methods will likely result in higher-quality inferences than if the two forms of data analysis are distinct, unintegrated components of the research [34].

### Incorporating time in delivery-system research

#### Background and challenges

The concept of time introduces important complexities in estimating delivery-system effects, program and evaluation design, and measurement and analysis of patient change in delivery-system research. For example, interactions among a system-level intervention and individual outcomes may need to incorporate time as an analytic variable to address whether interventions are more effective in early stages of implementation or whether their effects attenuate over time. Assessment of time as a dimension embedded within individuals as growth trajectories may influence both the design of interventions and the analysis of the effects of the interventions. Patient growth trajectories are simply paths, progressions, or lines of development, typically in some outcome of interest (e.g., patient self-management of diabetes, clinical symptoms, regularity of provider visits). Learning about predictors of trajectories, for example, can help to inform interventions (as well as policy) by suggesting when to introduce interventions or determining the expected direct or joint effects of the intervention and time.

In a similar fashion, organizations, teams, or other higher-level entities may also exhibit growth trajectories as a function of factors such as learning, history, or size. For example, at the environmental and organizational levels, interventions that change structures or processes often play out over longer time intervals in some organizations than in others, and not always in linear fashion. Hence, defining and measuring the change period/duration of effects, building appropriate lags between change introduction and expected effects, and understanding key sequences and/or time ordering of steps involved in delivery-system change are all crucial in delivery-system intervention design and evaluation.

### Recommendations: Methods for incorporating time in delivery-system research

Factors such as healthcare markets, societal norms and beliefs, changes in national and state policy, and local environmental and organization factors can all influence an intervention's implementation, sustainability, and effectiveness. This is particularly important because the effects of an intervention on individual outcomes may vary over time as a function of the uptake of particular program elements, entropy of those elements, or exogenous changes in organizational contexts [35]. Greater recognition of the potential for a planned intervention to deviate from the intended design requires researchers to more closely focus on identifying essential (i.e., active) program components and monitoring the extent to which the treatment protocol is adhered to or modified in practice and over time [29]. This implies an explicit, longitudinal assessment of key aspects of the intervention (presumably guided by treatment theory). Thus, time should be viewed as an important analytic concept in its own right, not simply as an element of the research design. For example, research on new models of care delivery (e.g., chronic care model, PCMH) suggests that implementation does not proceed at the same pace for different model components (e.g., shared goal setting with patients, use of patient registry for population health management) [25]. Under such circumstances, where different components may be implemented at different rates, more explicit consideration of time and sequencing (e.g., direct measurement of event occurrence), beyond being simple features of the research design, will be required to elucidate these types of issues and their consequences for effectiveness.

In a related vein, delivery-system researchers need to incorporate into their models temporal aspects of the relationship between the intervention and expected effects on patient outcomes. Such temporal effects may be manifest at multiple levels. For example, the time period over which systems change is expected may be directly influenced by implementation of the intervention at multiple contextual levels (i.e., organizational, policy, system). If the impact on outcomes is mediated by more pervasive organizational system effects, this impact may lag in time and only be observable after the program or evaluation ends. Obviously, this situation creates problems for assessing intervention effects over a short study time frame. Conversely, attributing causality to the program or intervention over longer time frames is subject to concurrent influences, or secular changes, or events that may confound any intervention effect. Collins and Graham, for example, caution that a mismatch between measurement intervals and anticipated outcomes may impair a researcher's ability to correctly model the processes of interest [36].

At the individual patient level, researchers should consider three approaches for incorporating time and growth trajectories in delivery-system models. The first approach is to identify temporal patterns in the data. For example, does the outcome increase, decrease, or remain stable over time? Is the general pattern linear or nonlinear? Are there abrupt shifts at substantively interesting moments? Second, researchers should directly assess time by including time-varying predictors (i.e., variables whose values vary over time). These can be used to identify, for example, whether patient participation in a delivery-system intervention varies over time. Alternatively, time-varying predictors can be used to model how changes in factors such as family circumstances (e.g., income, social support) or organizational policies may influence the intervention's effects and the conditions influencing these effects. The third approach, which is perhaps the most important for purposes of delivery-system change, is to examine interactions among intervention variables and patient growth trajectories. This approach will test whether an intervention's effect on patient outcomes varies over time [37]. This is particularly important, given that some effects dissipate over time; some effects increase, for example, as individuals become acclimated to the procedure or condition, and some effects may be especially pronounced at particular times. Note, however, that the last approach will place considerable demands on sample size (program, organization, system), as well as patient panel size.

Incorporating time as a component in delivery-system research often necessitates longer study periods, greater costs, and methodological challenges, particularly if multiple measurement points are involved. For example, in designing delivery-system evaluations, at least three outcome measurement points for a cohort of subjects are necessary to allow estimation of individual growth trajectories [38]. Other time-related challenges include attrition, loss to follow-up, and greater data collection and administrative burden on sites implementing the intervention.

However, incorporating time into study designs in delivery-system research also has analytic advantages. For example, the power to estimate group-to-group variability and group-level effects is strongly dependent on the number of groups included in the analyses. Failure to observe significant group-to-group variability is a common occurrence in delivery-system research owing to small sample sizes at higher levels of analysis, such as healthcare teams, organizations, or local healthcare markets. However, this should not always be taken as an indication that groups can be ignored in the analyses. Including a time dimension to assess group or any higher-level factors will likely increase the statistical power of the analysis owing to the addition of multiple observations/measurement points for any given group, thereby effectively increasing sample size and statistical power.

### General recommendations and conclusions

Consistent with the tenets of our system theory framework, many of the salient issues in delivery-system research are interrelated, not independent. For example, the challenges of how to deal with time in delivery-system research also has implications for assessing context and specifying appropriate lags between intervention introduction and outcomes assessment. Similarly, measuring readiness for change may have implications for sample selection, comparisons across intervention sites, or generalization of findings. Whereas addressing these issues piecemeal may result in small marginal improvements in the quality of research, it will likely take a multifaceted approach to show real gains. Indeed, given the systems perspective that is increasingly advanced by implementation and delivery-system researchers, failure to draw upon the rich set of research tools available will likely continue to impede our understanding of how and why interventions affect outcomes and, thus, how these interventions can be leveraged in the most effective ways in different settings. However, such multifaceted, systems-oriented approaches will challenge our current linear thinking about how systems of care delivery work and the traditional "internal validity at all costs" frameworks for designing and conducting research on delivery-system change.

In attempting to address the issues outlined in this paper, we need not abandon research methods such as randomized control trials and quasi-experimental designs that emphasize internal validity. These are and will continue to be critically important to furthering efforts in delivery-system research. However, these traditional approaches should be complemented by and integrated with methods that specifically address the dynamic and systemic qualities of most delivery-system changes.

The burden for implementing these recommendations, however, does not fall solely on investigators. Funding agencies should consider supporting studies with larger organizational sample sizes to allow appropriate modeling of contextual effects and assess the generalizability of interventions. Also, studies of longer duration should be funded to permit assessment of fidelity and sustainability of delivery-system change, as well as growth trajectories in individual patient outcomes. Such studies are important for improving our knowledge about the intermediate and long-term effectiveness of interventions and whether resources should be dedicated to disseminating these interventions more broadly. Consideration should also be given to funding retrospective assessments of previously funded interventions to assess these issues. Finally, funding agencies should encourage applications that employ more nontraditional, mixed-methods designs to advance understanding of the "why" and "how" questions of delivery-system changes and their effects. Answers to such questions speak to issues of how to improve interventions, as well how widely they might be disseminated. In sum, without the support and direction from these funding agencies, our knowledge about health service interventions and delivery-system change, much like the quality of the research itself, seems poised to improve only marginally.

For each of these delivery-system research challenges, the appropriate analytic and methodological tools have developed at a faster pace than our ability to apply them in an ordered, systematic manner [39]. Indeed, there are major challenges with such efforts given the absence of robust conceptual frameworks to guide this work. The application of these techniques without clear theoretic guidance increases the risk of inappropriately generalizing findings to other settings, omitting variable bias, or conducting studies where each investigation contributes little to our cumulative understanding of how to create more effective delivery systems. Given the added expense of longer study periods and larger sample sizes proposed in this paper, careful attention to such treatment theories becomes even more important. It is doubtful that a one size fits all approach will work for all interventions, classes of individuals, or types of illnesses. However, a more refined understanding of the mechanisms underlying delivery-system interventions (treatment theory) and the way in which outcomes for different classes of individuals change over time (growth trajectories) are fundamental starting points for capturing the heterogeneity in samples of individuals exposed to the intervention [40,41]. As theoretical development in these areas improves, inefficiencies will likely be reduced. However, in the near term, we can expect to incur significant costs as empirical work and theoretical development proceed unevenly. Our collective challenge in delivery-system research is to bridge this gap in order to accelerate the development of a cumulative body of knowledge and evidence on delivery-system change, rather than a set of unrelated, idiosyncratic studies.

## Notes

^1^This paper is based on a white paper commissioned by the Agency for Healthcare Research and Quality (AHRQ). The issues identified in the paper are based on the authors' knowledge of the implementation and delivery-system literatures, past empirical research that has examined the intersection of these literatures, as well as input from participants (AHRQ grantees, AHRQ staff, and health services research experts) of a meeting that was convened to identify and prioritize methods and metrics issues to be addressed in the next generation of delivery-system research. More details of this meeting, including other presentations, can be found at http://www.ahrq.gov/qual/deliverysys/2011mtg/.

## Competing interests

The authors declare that they have no competing interests.

## Authors' contributions

JA wrote the initial draft and assumed responsibility for reviewing content in the final version of the manuscript. LH took the lead in reorganizing content material in response to reviewers comments and providing examples of key challenges in delivery system research. All authors read and approved the final manuscript.
